# Methane Concentration Inversion Based on Multi-Feature Fusion and Stacking Integration

**DOI:** 10.3390/s25071974

**Published:** 2025-03-21

**Authors:** Yanling Han, Wei Li, Congqin Yi, Ge Song, Yun Zhang

**Affiliations:** Shanghai Marine Intelligent Information and Navigation Remote Sensing Engineering Technology Research Center, Key Laboratory of Fisheries Information, Ministry of Agriculture, College of Information, Shanghai Ocean University, Shanghai 201306, China; ylhan@shou.edu.cn (Y.H.); 18035494218@163.com (W.L.); gsong@shou.edu.cn (G.S.); y-zhang@shou.edu.cn (Y.Z.)

**Keywords:** methane concentration, feature fusion, Stacking ensemble, eastern Xinjiang, seasonal variation

## Abstract

To address the issue of relatively simple features and methods used in methane concentration inversion, which leads to low overall accuracy, this study proposes a methane concentration inversion method based on multi-feature fusion and Stacking ensemble learning. The method leverages the series-parallel cascade structure between multiple base models and meta-models to learn different feature representations and patterns in the original data, fully exploring the intrinsic relationships between various feature factors and methane concentration. This approach improves inversion accuracy and generalization capability. Finally, the research team conducted experimental validation in the eastern region of Xinjiang. The experimental results show that, compared with other typical methods, the Stacking ensemble model proposed in this study achieves the best inversion performance, with R^2^, RMSE, and MAE values of 0.9747, 2.8294, and 1.5299, respectively. In terms of seasonal distribution, methane concentration in eastern Xinjiang typically shows lower average values in the spring and autumn and higher average values in the summer and winter.

## 1. Introduction

Methane (CH_4_) is the second most significant greenhouse gas after carbon dioxide (CO_2_). Existing research indicates that the increase in atmospheric methane concentration is largely driven by continuously rising anthropogenic emissions [1,2]. Currently, the primary anthropogenic sources of methane include fossil fuel extraction and use, ruminant emissions, biomass burning, and rice cultivation [3,4]. Since 1750, methane emissions have contributed to nearly one-quarter of the cumulative radiative forcing caused by greenhouse gases, playing a significant role in global warming [5,6]. Additionally, due to its efficient absorption of infrared radiation, methane has a higher global warming potential (GWP) than CO_2_, with a GWP 28 times that of CO_2_ over a 100-year period [7,8]. Methane is also an important precursor of ozone (O_3_) and can affect air quality through photochemical reactions [9]. In the stratosphere, methane is oxidized to form water vapor (H_2_O), which is eventually converted into CO_2_, further exacerbating global warming [10,11]. Therefore, methane concentration inversion is of great significance for assessing global warming, identifying major emission sources, and addressing climate change.

Currently, atmospheric methane concentration observation methods primarily include ground-based and satellite-based approaches. Ground-based observations mainly consist of the Global Atmosphere Watch (GAW) and the Global Greenhouse Gas Reference Network (GGGRN) [12,13,14,15,16]. Since the 1980s, China has established atmospheric background stations, including the Waliguan station in Qinghai, to monitor greenhouse gases such as methane [17]. Ground-based observations, through long-term and high-precision monitoring of atmospheric methane (CH_4_) concentrations, can reveal its spatial patterns, seasonal fluctuations, and interannual variations. However, due to the limited number of stations, small monitoring coverage, and uneven distribution, ground-based monitoring is constrained in its spatiotemporal coverage. In contrast, satellite remote sensing, with its advantages of rapid observation, stable cycles, and large-scale synchronous monitoring, has become an important tool for continuous global methane concentration monitoring [18,19,20,21].

In recent years, many scholars have used satellite remote sensing data and statistical methods to analyze the spatial and temporal characteristics of global atmospheric methane concentration. Zhang Shaohui et al. [22] used AIRS satellite data and statistical methods to analyze the spatiotemporal variations and seasonal changes in methane concentration in the global and East Asian regions from December 2002 to November 2016. The study found that the global average methane concentration increased year by year from 2003 to 2016; the methane concentration in the East Asian region showed seasonal changes. He Qian et al. [23] used SCIAMACHY satellite remote sensing data and statistical methods to analyze the changes in global methane concentration from 2003 to 2009. The study found that southern China, India, and the Southeast Asian peninsula are high-value areas for global methane concentration. At the same time, latitude has an impact on methane concentration, which gradually decreases from north to south. Li Shengwei et al. [24] used methane observation data from the TROPOMI satellite and wind data from the ECMWF global reanalysis to estimate surface methane emissions in China using an efficient divergence method. The results showed that the high-concentration areas of methane were in central China, East China, the Beijing–Tianjin–Hebei region, the Sichuan Basin, and some northern parts of the Xinjiang Uygur Autonomous Region.

Existing research on methane concentrations mainly relies on statistical models and mechanistic models. Statistical models, while capable of providing certain trend analyses, often lack an in-depth understanding of physical and chemical mechanisms. Mechanistic models, on the other hand, require comprehensive consideration of complex physical and chemical processes, increasing model complexity [25,26]. In addition, dependence on specific environments and assumptions about boundary conditions also affect the applicability of these models in practical use. Therefore, these traditional models often show limitations when dealing with complex nonlinear relationships and multi-source data fusion. In contrast, machine learning methods can learn complex nonlinear relationships from large amounts of data, exhibiting significant advantages such as high accuracy and strong adaptability. They provide an efficient and flexible solution for monitoring atmospheric methane concentrations and have proven effective in fields such as simulating atmospheric pollution [27,28].

In recent years, machine learning has demonstrated exceptional performance in methane concentration monitoring. Xinyue Ai [4] utilized TROPOMI satellite methane concentration data and anthropogenic emission inventories to analyze the methane concentration enhancement trends in central and eastern China from 2001 to 2018 using the random forest method. The study found that the random forest model could accurately establish the relationship between emission sources and methane concentration enhancement, with a coefficient of determination (R^2^) of 0.89 and a root mean square error (RMSE) of 11.98. Guo Haohao et al. [29] employed GOSAT satellite methane concentration data and applied the random forest method to establish the relationship between various influencing factors and methane concentrations. The results indicated that the spatial distribution of near-surface methane concentrations in China generally exhibited higher levels in the east and lower levels in the west. Seasonally, methane concentrations were generally higher in summer and autumn and lower in spring and winter.

The above research demonstrated the potential of machine learning methods in monitoring atmospheric methane concentrations, but there were still some limitations. Most existing studies relied on single features or limited data sources, such as using only meteorological data or specific satellite data. This use of single features restricted the model’s ability to fully understand the complexity of methane concentration changes, making it difficult to fully capture the inherent relationships between methane concentrations and various factors. Additionally, single-feature models often struggled to adapt to the complex spatiotemporal variations, making it challenging to address the changing patterns of methane concentrations across different regions and seasons.

To address the above issues, this study proposed a methane concentration inversion method based on multi-feature fusion and the Stacking ensemble model (MFF-SEM). It used Level 2 methane column concentration data from the TROPOMI instrument on Sentinel-5P as the research object, combined with the ERA5 dataset provided by the European Centre for Medium-Range Weather Forecasts (ECMWF) and auxiliary data such as latitude and longitude. By comprehensively considering meteorological factors and other characteristic parameters that affect methane concentration, this study constructed a stacked ensemble learning methane inversion model based on multi-feature fusion. The model integrated multiple base models and a meta-model to capture different feature representations and patterns in the original data through cascaded learning, achieving complementary advantages and multi-feature fusion. This approach fully explored the hidden relationships between different features and methane concentration, enabling accurate methane inversion and spatiotemporal variation analysis. The method was validated through experiments in the eastern Xinjiang region, demonstrating its effectiveness.

## 2. Materials and Methods

### 2.1. Study Area Description

Xinjiang is located in the hinterland of the Eurasian continent and is characterized by a temperate continental climate. The annual average temperature is 32 °C, and the annual average precipitation is approximately 150 mm, indicating a dry climate [30]. The study area covers the eastern part of Xinjiang, with a longitude range of 84°29′4″ E to 94°9′18″ E and a latitude range of 37°59′42″ N to 46°11′6″ N. The study period spanned from May 2018 to May 2020.

### 2.2. Data Collection and Processing

This study combines meteorological factors, satellite auxiliary data, and latitude–longitude information to invert methane concentrations.

Research has shown that incorporating meteorological data into modeling can enhance the accuracy and reliability of methane concentration inversion. The meteorological factors used in this study were derived from the ERA5 dataset provided by the European Centre for Medium-Range Weather Forecasts (ECMWF). This dataset assimilates conventional observations and satellite remote sensing data from the surface and upper atmosphere across different regions globally [31,32,33]. As shown in Table 1, the meteorological factors employed in this study include 10 m zonal wind (u10), 10 m meridional wind (v10), 2 m temperature (t2m), 2 m dewpoint temperature (d2m), surface direct solar radiation under clear sky (cdir), near-infrared albedo for diffuse radiation (alnid), surface pressure (sp), surface solar radiation (ssr), total column ozone content (tco3), and boundary layer height (blh). The data have a spatial resolution of 0.25° × 0.25° and a temporal resolution of hourly.

The auxiliary data used in this study come from the accompanying data of the Tropospheric Monitoring Instrument (TROPOMI) L2 product output, which can be used for a posteriori filtering and total inversion error estimation, and can also be used for model analysis. The auxiliary data selected in this study include total column water vapor (water_total_column), aerosol optical thickness (aerosol_optical_thickness) and surface albedo (surface_albedo). The spatial resolution of the auxiliary data is 7 km × 7 km, and the temporal resolution is daily. In addition to the above auxiliary data, the features involved in the modeling also include longitude and latitude to characterize spatial characteristics.

The methane concentration data come from the methane (CH_4_) column concentration product of the Tropospheric Monitoring Instrument (TROPOMI). TROPOMI, an atmospheric monitoring spectrometer onboard the Sentinel-5P satellite, is one of the most technologically advanced and highest-spatial-resolution atmospheric spectrometers globally, providing daily global coverage [34,35]. The global spatial resolution is 7 km × 7 km (improved to 5.5 km × 7 km in August 2019). The CH_4_ column concentration product is a Level 2 (L2) offline (OFFL) data product. For this study, high-quality CH_4_ column concentration inversion data, after bias correction (methane_mixing_ratio_bias_corrected) and with a quality descriptor (qa_value > 0.5), are selected.

**Figure 1:**
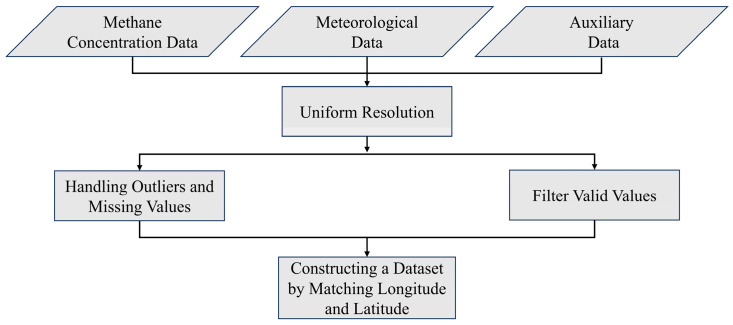
Data processing procedure.

The data are processed from both temporal and spatial aspects. As shown in Figure 1, at the spatial scale, to eliminate the scale effect of different resolution data, meteorological factors are resampled to 7 km × 7 km resolution using bilinear interpolation method. At the temporal scale, to minimize errors, the values of meteorological factors closest to the methane concentration time are used. For anomalies and missing values, this study directly removes the missing values, and anomalies are processed using box plots. In order to guarantee the highest-quality data for methane concentration data, pixels classified with a qa_value of less than 0.5 are filtered to guarantee data quality.

### 2.3. Stacking Ensemble Learning Model for Methane Concentration Inversion

The core idea of Stacking ensemble learning is to combine multiple base learning models, leveraging the strengths of different models to capture patterns and relationships that might be overlooked by individual models, thereby reducing model bias and variance. A meta-model is then used to integrate the predictions of the base models, enhancing the overall generalization capability of the model. In this study, GBDT and LightGBM are employed to fit residuals through gradient boosting algorithms, effectively capturing the nonlinear feature mapping between different features and methane concentrations. Additionally, XGBoost and RF are utilized for their exceptional performance in preventing model overfitting. This approach achieves complementary advantages among the base models, fully exploring the intrinsic relationships between features and methane concentrations. Finally, the meta-learner Lasso is used to achieve precise inversion of methane concentrations.

**Figure 2:**
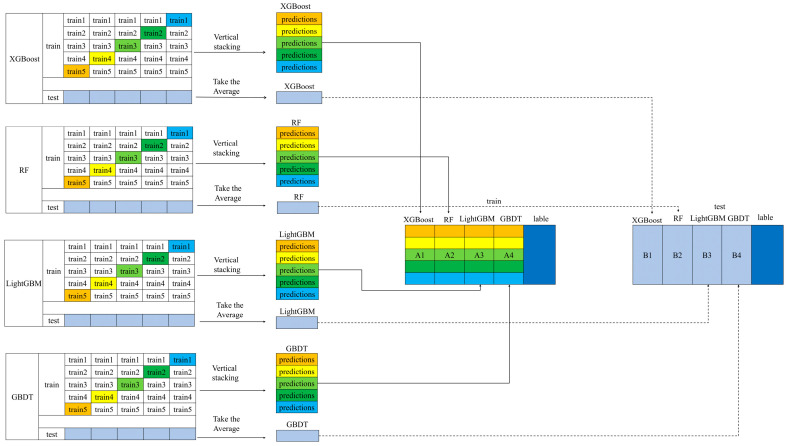
Framework diagram of Stacking ensemble learning.

Figure 2 shows the framework of methane concentration inversion based on Stacking ensemble learning. In this study, five-fold cross-validation is used to train the base learners. The training set is divided into five parts, with four parts used for training and the remaining one part, along with the test set, used for prediction. This process is repeated five times, and the prediction results for each sample are concatenated in their original order to generate a new feature matrix for the meta-learner training set. Simultaneously, the prediction results from the five test sets are averaged to create a new feature matrix for the meta-learner test set.

### 2.4. Base Learner

GBDT (Gradient Boosting Decision Tree) is an iterative decision tree algorithm that is part of the boosting strategy and comprises multiple decision trees. As a supervised machine learning algorithm, GBDT can accurately capture the nonlinear characteristics of various predictive variables and establish the relationship between predictors and methane concentrations. Its core idea is to use the gradient boosting method, where each iteration adds a new decision tree based on the previous one to fit the residuals between the predicted values and the true values from the last iteration [36]. In this way, each iteration brings the predicted results closer to the true values, and the optimal prediction is ultimately obtained by accumulating the results of multiple decision trees. The calculation formulas are shown in Equations (1)–(4):

(1) $$f_{0}\left(x\right)=argmin\sum_{i=1}^{n}L(y_{i},f(x))$$

(2) $$f_{t}\left(x\right)=f_{t-1}\left(x\right)+\Delta f_{t}\left(x\right)$$

(3) $$\;\Delta f_{t}\left(x\right)=P_{t}h_{t}$$

(4) $$F(x)=\sum_{t=0}^{T}f_{t}\left(x\right)$$

In the formula, $f_{t}\left(x\right)$ represents the function after the *t*-th iteration, $L(y_{i},f(x))$ denotes the loss function, $\Delta f_{t}\left(x\right)$ is the boost value after each iteration, ${Ρ}_{t}$ is the optimal gradient descent step length, $h_{t}$ is the base-learner function, and $F\left(x\right)$ is the total boost value after iteration.

XGBoost, a machine learning algorithm, is characterized by its extensive use, flexibility, and efficiency. It is based on the gradient boosting framework and improves prediction accuracy by integrating multiple decision tree models. The core idea is that during the training process, each new decision tree is constructed to improve upon the results of the previous tree, aiming to gradually reduce the loss function value and thereby enhance the predictive capability of the entire model [37]. The loss function is defined as follows:

(5) $$L=\sum_{i=1}^{n}l(y_{i},{\hat{y}}_{i})$$

In the equations, $y_{i}$ represents the true value of methane concentration, ${\hat{y}}_{i}$ denotes the predicted value of methane concentration, and n is the number of samples. To prevent overfitting, XGBoost introduces a regularization strategy into the objective function. It controls the complexity of the decision trees to limit the model’s learning capacity. The objective function is defined in Equation (6):

(6) $$Obj=\sum_{i=1}^{n}l(y_{i},{\hat{y}}_{i})+\sum_{i=1}^{t}\Omega (f_{i})$$

The regularization term, which sums the complexity of all trees, is incorporated into the objective function to prevent overfitting. Commonly used regularization strategies include limiting the maximum depth of decision trees, the minimum number of samples in leaf nodes, and the weight decay of leaf nodes.

LightGBM is a gradient boosting algorithm based on Gradient Boosting Decision Trees (GBDTs). Compared to traditional gradient boosting methods, LightGBM significantly improves training speed and efficiency by introducing Exclusive Feature Bundling (EFB) and a histogram-based algorithm. It adopts a leaf-wise optimization strategy, which effectively reduces memory usage and demonstrates excellent performance on large-scale datasets and high-dimensional features.

Random forest (RF) is a machine learning algorithm based on the Bagging strategy, which can effectively handle nonlinear problems and excels at processing large numbers of samples and features. It constructs multiple decision trees by randomly sampling a subset of the original data with replacement and then aggregates their results through voting or averaging to obtain the final prediction [38]. By combining different models, this technique effectively prevents overfitting while strengthening the model’s generalization ability.

### 2.5. Meta-Learner

The Lasso regression model is a linear regression method that achieves feature selection and model simplification by introducing an L1 regularization term. Its core idea is to constrain the model coefficients through the L1 regularization term $\lambda {\left|\right|\beta \left|\right|}_{1}$, which forces the coefficients of less important features to become zero, thereby enabling feature selection. This approach not only reduces model complexity and improves generalization capability but also enhances model interpretability. It is shown in Equation (7):

(7) $$\underset{\beta }{\min}\left\{{\left|\right|y_{t}-\beta x_{t}\left|\right|}_{2}^{2}+\lambda {\left|\right|\beta \left|\right|}_{1}\right\}$$

where $y_{t}$ and $x_{t}$ are the *t*-th response variable and independent variable, respectively, and β is the parameter vector.

### 2.6. Model Evaluation Metrics

To evaluate model performance, this study uses the coefficient of determination (R^2^), root mean square error (RMSE), and mean absolute error (MAE) as performance metrics. The calculation formulas are shown in Equations (8)–(10):

(8) $${\;R}^{2}=1-\frac{\sum_{i=1}^{m}{\left({\hat{y}}_{i}-y_{i}\right)}^{2}}{\sum_{i=1}^{m}{\left({\overset{-}{y}}_{i}-y_{i}\right)}^{2}}$$

(9) $$RMSE=\sqrt{\frac{1}{m}\sum_{i=1}^{m}{\left({\hat{y}}_{i}-y_{i}\right)}^{2}}$$

(10) $$MAE=\;\frac{1}{m}\sum_{i=1}^{m}\left|{\hat{y}}_{i}-y_{i}\right|$$

where $y_{i}$ represents the actual methane concentration value of the *i*-th sample; ${\hat{y}}_{i}$ represents the predicted methane concentration value of the *i*-th sample; ${\overset{-}{y}}_{i}$ represents the mean value of actual methane concentrations; *m* represents the number of measurements. R^2^ measures the correlation between model predictions and actual values, with values close to 1 indicating strong correlation. RMSE emphasizes prediction error magnitude and model stability, where lower values indicate higher model stability. MAE reflects the overall degree of prediction error, where lower values indicate more accurate model predictions.

## 3. Results and Discussion

### 3.1. Experimental Setup

In this study, a multi-feature fusion Stacking ensemble model (MFF-SEM) is established using four machine learning models including XGBoost as base learners and the Lasso model as the meta-learner. The partial model parameters are shown in Table 2.

### 3.2. Factor Selection for MFF-SEM Model

To demonstrate the rationality of feature factor selection in this study, comparative experiments with different feature combinations are conducted. As shown in Table 3, F1, F2, and F3 represent three different feature combinations, where F1 represents meteorological factor features only; F2 represents meteorological factors and auxiliary data; and F3 represents all features including meteorological factors, auxiliary data, and geographical coordinates.

**Figure 3:**
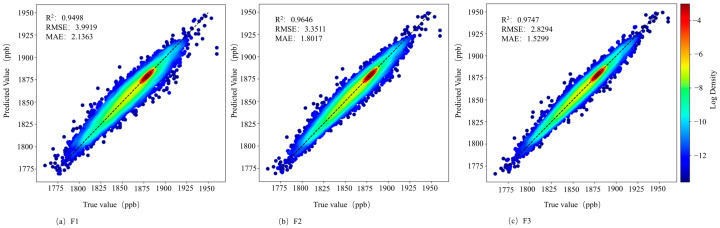
Experimental results of different feature combinations.

The data from May 2018 to April 2019 are used, with 80% as the training set and 20% as the test set, and the final results are shown in Figure 3. Figure 3a–c represent the results using F1 meteorological factors only, F2 meteorological features with auxiliary data, and F3 all feature combinations, respectively. The results indicate that when using only meteorological factors as features, the MFF-SEM model shows the lowest accuracy, with a coefficient of determination (R^2^) of 0.9489. The highest accuracy is achieved using all feature combinations, with R^2^ of 0.9747, RMSE of 2.8294, and MAE of 1.5299. The results also demonstrate that the model using all feature combinations performs better than the other two models in terms of fitting effect. These results confirm that the complete feature combination selected in this study has significant advantages.

**Figure 4:**
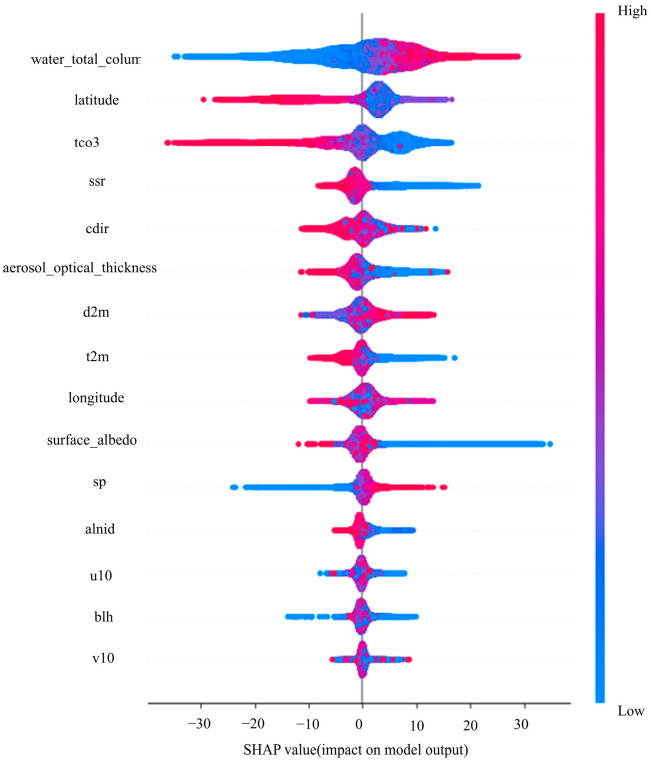
SHAP summary plot.

To investigate factors influencing methane concentration more accurately, SHAP (SHapley Additive exPlanations) values are used to analyze the magnitude and direction of feature impacts on model predictions. Figure 4 presents the SHAP summary plot, which ranks the importance of selected factors affecting methane concentrations. Each point in the plot represents a sample, and each row represents a feature. The SHAP values are centered at zero, where samples on the left side have negative effects on predictions, and those on the right side have positive effects. The colors indicate the magnitude of corresponding feature values, transitioning from red to blue as feature values decrease. The wider the color region, the greater the feature’s influence.

As shown in Figure 4, temperature (t2m) has a negative impact on methane concentration, where higher temperatures correspond to lower SHAP values and lower methane concentrations in the corresponding regions. Boundary layer height (blh) and 10 m zonal wind (u10) also exhibit negative effects on methane concentration. Higher boundary layer height leads to a larger methane dispersion range, resulting in lower concentrations. Additionally, increased wind speed enhances long-distance transport and diffusion of airflow, which also reduces methane concentration.

**Figure 5:**
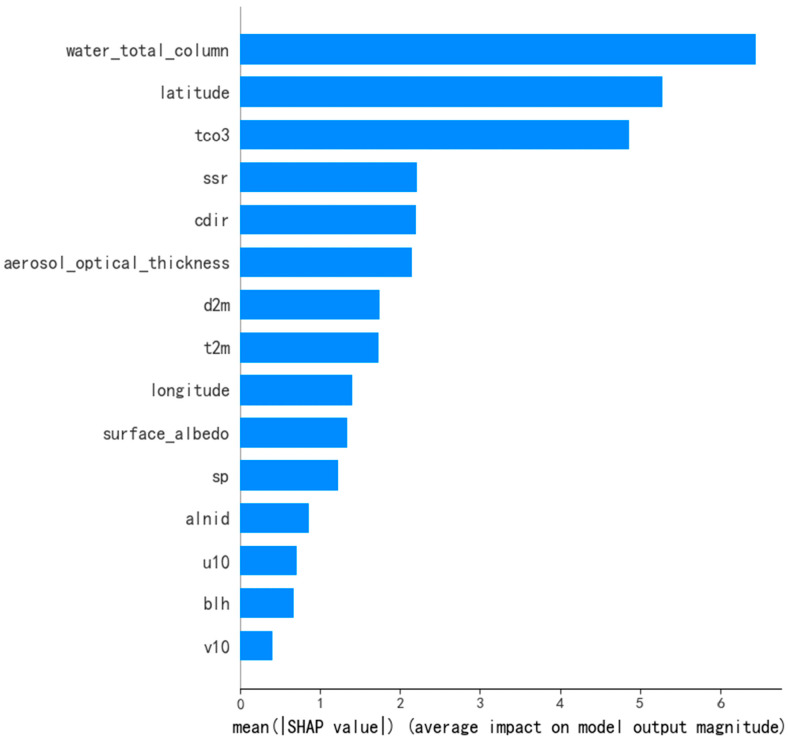
SHAP feature importance plot.

Figure 5 shows the SHAP feature importance plot, where the global importance of each feature is calculated by averaging the absolute values of SHAP values. As shown in the figure, the total column water vapor (water_total_column) and latitude (latitude) from the auxiliary data, as well as the total column ozone (tco3) from the meteorological factors, have a significant impact on the methane concentration inversion model. This also indicates the importance of the combination of these three features for model construction.

### 3.3. Accuracy Analysis and Comparison Between Stacking Model and Other Single Models

The data from May 2018 to April 2019 are used, with 80% as the training set and 20% as the test set. The data are input into the MFF-SEM model and other models for experimentation, and the final model results are shown in Table 4.

**Figure 6:**
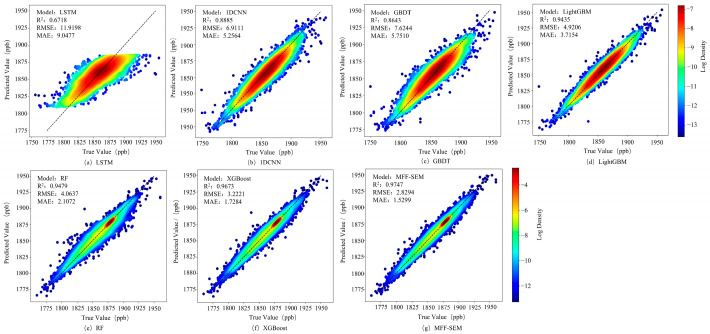
Density scatter plots of actual values versus predicted values for different models.

From the comparison of evaluation metrics for different models in Table 4, it can be observed that deep learning models generally exhibit lower accuracy. Due to the limited sample size, the LSTM model struggles to effectively capture temporal dependencies, resulting in lower inversion accuracy, with R^2^, RMSE, and MAE values of 0.6718, 11.9198, and 9.0477, respectively. The 1DCNN model requires a larger dataset, and the limited data volume in methane concentration inversion affects its performance, making it only slightly better than the GBDT model among machine learning models (R^2^ of 0.8643). Machine learning models such as LightGBM, RF, and XGBoost generally outperform deep learning models in terms of accuracy. Among them, LightGBM and RF show similar performance, with R^2^, RMSE, and MAE values of 0.9435, 4.9206, 3.7154 and 0.9479, 4.0637, 2.1072, respectively. The XGBoost model, which incorporates a regularization term to effectively prevent overfitting, achieves higher inversion performance, with R^2^, RMSE, and MAE values of 0.9673, 3.2221, and 1.7284, respectively. However, the MFF-SEM model used in this study, by leveraging multiple base learners to fully explore the relationships between different feature factors and methane concentrations, achieves deep integration of feature combinations and complementary advantages among base learners, resulting in the highest inversion accuracy, with R^2^, RMSE, and MAE values of 0.9747, 2.9294, and 1.5299, respectively. Figure 6 shows density scatter plots of actual values versus predicted values for different models. 

Figure 6a–g represent LSTM, 1DCNN, GBDT, LightGBM, RF, XGBoost, and MFF-SEM models, respectively. The results indicate that Stacking, XGBoost, and RF models show similar data distributions, with concentration values primarily distributed in the 1875–1900 ppb range. Among these, the MFF-SEM model demonstrates the best fitting performance, while XGBoost and RF models underestimate methane concentrations. LightGBM and GBDT models predict concentration values concentrated between 1810 and 1910 ppb, with LightGBM showing better fitting performance than GBDT. GBDT exhibits more outliers, mainly due to its sensitivity to anomalous values, which leads to decreased performance. Among deep learning models, LSTM shows the poorest fitting performance, with predicted concentration values concentrated between 1820 and 1880 ppb. For methane concentrations outside this range, the model predictions remain constant, primarily due to the imbalanced distribution of low and high concentration values in the samples. The limited sample size results in weak temporal relationships, preventing the model from learning the characteristics of these samples.

### 3.4. Seasonal Analysis of Methane Concentrations

**Figure 7:**
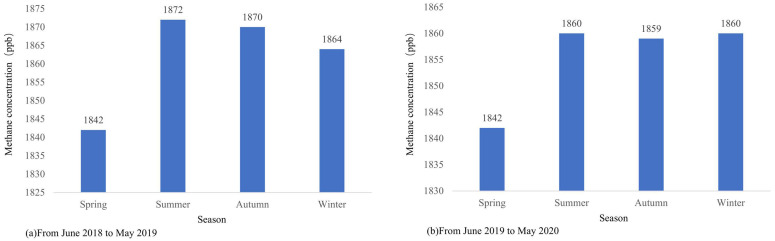
Seasonal average methane concentrations.

**Figure 8:**
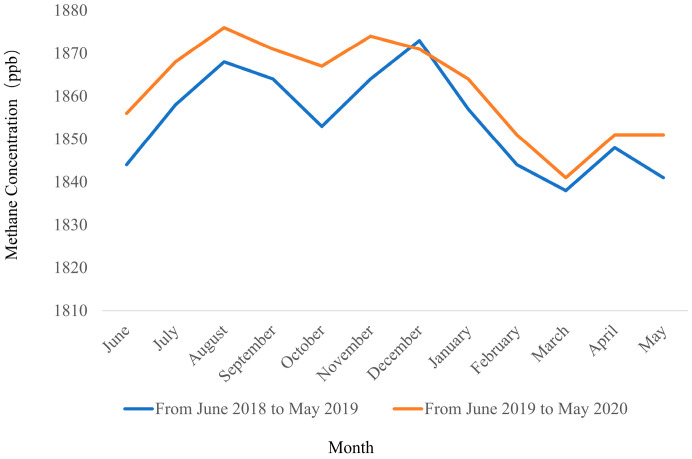
Monthly average methane concentration.

Figure 7 shows the seasonal average methane concentrations from June 2018 to May 2020, where Figure 7a represents the period from June 2018 to May 2019 and Figure 7b represents the period from June 2019 to May 2020. Figure 8 presents the monthly average methane concentrations. Both figures indicate that methane concentrations exhibit distinct seasonal variation characteristics and an upward fluctuating trend, with generally higher concentrations in summer and winter seasons and lower concentrations in spring and autumn seasons.

Among the four seasons, spring (March–May) shows generally lower methane concentrations due to lower temperatures and reduced natural and anthropogenic methane emissions. March exhibits the lowest concentrations, after which levels gradually begin to rise. In summer (June–August), methane concentrations start to increase rapidly from June, reaching their peak in August. This summer surge in methane concentrations is primarily attributed to accelerated plant growth promoted by high temperatures, increased emissions from vegetation such as rice paddies, and substantial methane contributions from natural water bodies like rivers and lakes. Additionally, high-temperature decomposition of urban waste and industrial emissions significantly increase methane sources. In autumn (September–November), as temperatures gradually decrease, microbial activity weakens, slowing methane generation, though mean concentrations maintain relatively high levels. In winter (December–February), methane concentrations show relatively high trends due to heating demands, which involve extensive fossil fuel extraction, transportation, and combustion emissions containing high methane levels. Furthermore, winter climate conditions favor methane accumulation.

### 3.5. Generalization Experiment

Section 3.3 confirms the superior performance of the MFF-SEM model in methane concentration inversion. To validate the model’s generalization ability, data from May 2018 to April 2019 are used as the training set, while data from May 2019 to April 2020 are used as the test set for experimentation.

From the comparative analysis results shown in Table 5, it can be observed that the performance of deep learning models is relatively poor, with R^2^ values of 0.4300 and 0.4059 for LSTM and 1DCNN, respectively. This is primarily due to the limited data volume of one year and the increasing trend of methane concentrations over time. Deep learning models require large amounts of data to effectively learn sufficient features and patterns, which leads to their poor generalization performance. Compared to other machine learning models, LightGBM, which incorporates techniques such as histogram-based algorithms and leaf-wise growth strategies, significantly improves training speed and prediction efficiency while reducing memory usage. It also demonstrates better generalization performance while enhancing model accuracy, with R^2^, RMSE, and MAE values of 0.5715, 12.1671, and 9.9433, respectively. The MFF-SEM model used in this study integrates the advantages of multiple base models, enabling it to fully learn the hidden relationships between different features and methane concentrations, achieving the best generalization performance. Specifically, its R^2^ is 0.5838, RMSE is 11.9903, and MAE is 9.8294. Compared to LightGBM, the R^2^ is improved by 2.15%, while RMSE and MAE are reduced by 1.45% and 1.14%, respectively.

**Figure 9:**
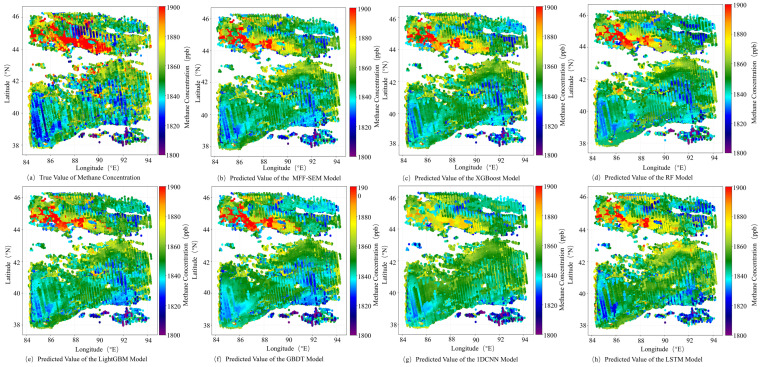
Different model predictions of methane concentration distribution.

As shown in Figure 9, the methane concentration inversion distribution maps of different models are displayed. Figure 9a shows the true values of methane concentrations from May 2019 to April 2020. Figure 9b–h present the extrapolation results of the MFF-SEM, XGBoost, RF, LightGBM, GBDT, 1DCNN, and LSTM models, respectively. The proposed MFF-SEM model achieves the best inversion results compared to other models, especially in regions with high methane concentrations, where its performance is significantly better. The XGBoost, RF, LightGBM, and GBDT models show similar results, while the LSTM and 1DCNN models perform poorly, generally underestimating methane concentrations.

Additionally, due to the seasonal characteristics of methane concentration variations, seasonal generalization experiments are also conducted in this study. Table 6 shows the extrapolation results for different seasons, where the training set consists of one full year of data from June 2018 to May 2019, and the test set comprises seasonal data from June 2019 to May 2020.

As shown in Table 6, the MFF-SEM ensemble learning model demonstrates the best overall inversion performance across all four seasons, with R^2^ values of 0.4733, 0.4755, 0.2534, and 0.6401 respectively. This superior performance is primarily attributed to Stacking’s effective integration of multiple base model predictions, which achieves complementary advantages and effectively reduces the bias and variance of single models, resulting in optimal inversion results. Furthermore, due to the limited sample size, which is unfavorable for deep learning models to fully learn the hidden patterns between features and methane concentrations, machine learning models show better overall inversion performance than deep learning models.

For different seasons, the MFF-SEM model exhibits stable generalization performance in spring, summer, and winter. However, all models perform poorly in autumn, which may be related to uncertain factors affecting methane emissions during this season. For example, autumn is accompanied by changes in agricultural activities, such as harvesting and straw burning, which increase methane emissions. The influence of these uncertainty factors reduces model performance.

**Figure 10:**
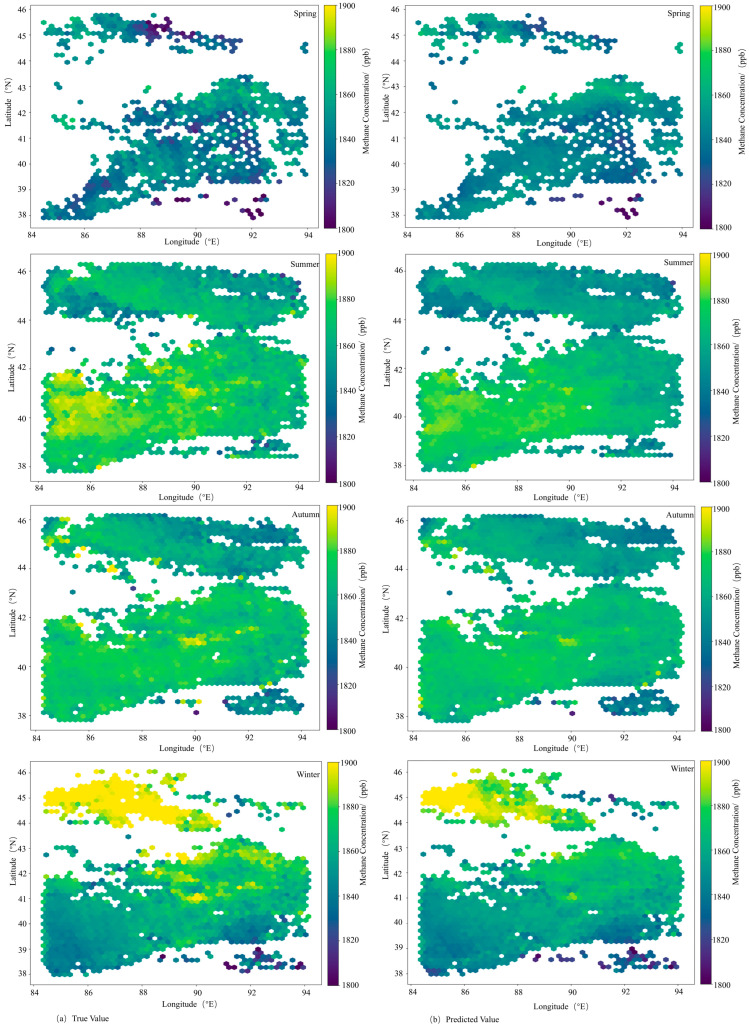
Comparison of true values and predicted values for the MFF-SEM.

Figure 10 shows the comparison between predicted and actual values of the MFF-SEM model for all four seasons, where Figure 10a represents actual values and Figure 10b represents predicted values. As shown in Figure 9, overall, the MFF-SEM model achieves satisfactory inversion results across spring, summer, autumn, and winter seasons. In spring, due to the influence of anomalous values, the model fails to fully capture the actual changes in methane concentrations, leading to overestimation by the MFF-SEM model. For other seasons, predicted methane concentrations are generally lower than actual values, primarily due to imbalanced sample distribution, where samples with high concentrations are relatively few, preventing the model from fully learning data characteristics and hidden associations.

## 4. Conclusions

To address the issues of single-feature selection and low accuracy in methane concentration inversion, this study proposes a methane concentration inversion method based on multi-feature fusion and the Stacking ensemble model (MFF-SEM). The method combines TROPOMI observation data with meteorological data, auxiliary data, and geographical coordinates. The research conducts experiments in the eastern region of Xinjiang. The experimental findings show that the proposed approach performs better overall compared to other common methane concentration inversion techniques. The following is a summary of the specific conclusions:

(1) The proposed MFF-SEM ensemble learning model effectively utilizes four base models (XGBoost, RF, LightGBM, and GBDT) and a Lasso meta-model in series-parallel cascade learning to capture different feature representations and pattern expressions from the original data. Through complementary advantages of multiple models, it thoroughly explores the intrinsic associations between multiple features and methane concentrations, achieving the best inversion performance with R^2^ of 0.9747, RMSE of 2.9294, and MAE of 1.5299.
(2) SHAP plot analysis reveals that total column water vapor (water_total_column), latitude, and total column ozone (tco3) make significant contributions to the model in methane concentration inversion. Features such as surface pressure (sp) show positive correlations with methane concentration variations, while features like 2 m temperature (t2m) and boundary layer height (blh) exhibit negative correlations with methane concentration changes.
(3) The mean methane concentrations from June 2019 to May 2020 are higher than those of the previous year, indicating an increasing trend in methane concentrations over the years. Summer methane concentrations are typically higher, primarily due to increased temperatures promoting methane generation and release. The decrease in methane concentrations in early 2020 is mainly attributed to reduced human activities during the pandemic. Overall, methane concentrations exhibit a pattern of higher levels in summer and winter, and lower levels in spring and autumn.
(4) In terms of extrapolation performance, the proposed MFF-SEM model also outperforms other models. The model achieves its best performance in winter, with an R^2^ of 0.6401. However, due to the influence of other complex factors, the inversion performance is relatively low in autumn. The overall lower extrapolation performance is primarily related to limited sample size and short time span. Future research will expand the study area and time span and incorporate physical models for in-depth investigation.

## Figures and Tables

**Table 1:** Data information and sources.

Name	Spatial Resolution	Temporal Resolution	Data Source
Meteorological features (u10, v10, t2m, d2m, cdir, alnid, sp, ssr, tco3, blh)	0.25° × 0.25°	1 h	ERA5 dataset provided by the European Centre for Medium-Range Weather Forecasts (ECMWF)
Auxiliary features	7 km × 7 km	1 d	Tropospheric Monitoring Instrument (TROPOMI)
CH_4_	7 km × 7 km	1 d	Tropospheric Monitoring Instrument (TROPOMI)

**Table 2:** Partial model parameters.

Model	Parameter Setting
XGBoost	n_estimators = 200 learning_rate = 0.01 max_depth = 10
GradientBoosting	n_estimators = 200 max_depth = 5 learning_rate = 0.1
Random Forest	n_estimators = 200 max_depth = 15
LightGBM	num_leaves = 200 max_depth = 30 learning_rate = 0.05
Lasso	alpha = 1.0 max_iter = 1500

**Table 3:** Three different feature combinations.

Feature Combination	Feature Description
F1	meteorological factors
F2	meteorological factors and auxiliary data
F3	meteorological factors, auxiliary data, and latitude and longitude

**Table 4:** Comparison of evaluation metrics for different models.

Model	R^2^	RMSE	MAE
LSTM	0.6718	11.9198	9.0477
1DCNN	0.8885	6.9111	5.2564
GBDT	0.8643	7.6244	5.7510
LightGBM	0.9435	4.9206	3.7154
RF	0.9479	4.0637	2.1072
XGBoost	0.9673	3.2221	1.7284
MFF-SEM	0.9747	2.8294	1.5299

**Table 5:** Comparison of model extrapolation evaluation metrics.

Model	R^2^	RMSE	MAE
LSTM	0.4300	14.0324	11.2621
1DCNN	0.4059	14.3266	11.5291
GBDT	0.5365	12.6542	10.2950
LightGBM	0.5715	12.1671	9.9433
RF	0.4538	13.7361	11.1318
XGBoost	0.5295	12.7493	10.3821
MFF-SEM	0.5838	11.9903	9.8294

**Table 6:** Prediction performance metrics for different seasons (R^2^).

Model	Spring	Summer	Autumn	Winter
1DCNN	0.2386	0.1224	−0.0794	0.5078
LSTM	0.3886	0.1234	0.1459	0.4343
RF	0.3302	0.3300	0.0146	0.5174
LightGBM	0.4150	0.4875	0.2262	0.6075
XGBoost	0.4301	0.3881	0.1518	0.6066
GBDT	0.4559	0.4065	0.1947	0.5650
MFF-SEM	0.4733	0.4755	0.2534	0.6401

## Data Availability

The data presented in this study are available on request from the corresponding author.
